# Biophysical Insight into the Interaction of Human Lysozyme with Anticancer Drug Anastrozole: A Multitechnique Approach

**DOI:** 10.1155/2020/8363685

**Published:** 2020-08-25

**Authors:** Fahad M. Almutairi, Mohammad Rehan Ajmal, Adel Ibrahim Al Alawy, Rizwan Hasan Khan, Ali Saber Abdelhameed

**Affiliations:** ^1^Physical Biochemistry Research Laboratory, Biochemistry Department, Faculty of Science, University of Tabuk, P.O. Box 741, Tabuk 71491, Saudi Arabia; ^2^Interdisciplinary Biotechnology Unit, Aligarh Muslim University, Aligarh, India; ^3^Faculty of Pharmacy, King Saud University, Riyadh, Saudi Arabia

## Abstract

In the present study, we employ fluorescence spectroscopy, dynamic light scattering, and molecular docking methods. Binding of anticancer drug anastrozole with human lysozyme (HL) is studied. Binding of anastrozole to HL is moderate but spontaneous. There is anastrozole persuaded hydrodynamic change in HL, leading to molecular compaction. Binding of anastrozole to HL also decreased in vitro lytic activity of HL. Molecular docking results suggest the electrostatic interactions and van der Waals forces played key role in binding interaction of anastrozole near the catalytic site. Binding interaction of anastrozole to proteins other than major transport proteins in blood can significantly affect pharmacokinetics of this molecule. Hence, rationalizing drug dosage is important. This study also points to unrelated effects that small molecules bring in the body that are considerable and need thorough investigation.

## 1. Introduction

Small ligands are known to intermingle with protein molecules readily [1, 2]. Protein-drug interaction studies are important and central in understanding biological processes. Recently, such studies are hot spots of multidisciplinary research [3, 4]. Drug binding to transport proteins can significantly affect metabolism of drug molecules. Proteins are versatile molecules and perform many different functions in the human body. Human lysozyme (HL) is a small globular protein. HL is found in secretions such as saliva and tears. It is a model protein [5, 6]. Ever since its discovery, lysozyme represented a prototype molecule for understanding the complexity of the protein structure and function [7]. Thus, the study on the interaction of drugs with lysozyme has important significance. Such studies are useful for providing information on the structural features of the proteins on interaction with drugs and to illuminate the therapeutic effectiveness of drugs [8, 9]. Human lysozyme (HL) is an important enzyme and is part of the body defense against many bacteria. HL is found in tears and saliva [10]. It is a single polypeptide comprising of seven helices and one beta sheet. There are no subunits or prosthetic groups. HL hydrolyze *β*-1,4-glycosidic linkages between N-acetylmuramic acid and N-acetylglucosamine; these linkages are present in the peptidoglycan cell wall of bacteria [11]. HL causes damage to the bacterial cell wall by degrading the polymer, leading to perforation and bacterial cell lyses [12]. The active site is easily visible in three-dimensional models. Hydrolysis of the cell wall of bacteria is an important component of antimicrobial action of HL [7]. Asp^53^ and Glu^35^ are main residues involved in activity [13] while many others are involved in holding ligands to active sites by hydrogen bonds. Lysozyme from different species seems to function in a similar way [14].

Anastrozole is an anticancer drug [15, 16] Anastrozole is also an important chemotherapy drug used in breast cancer after surgery to reduce the risk and used as hormone therapy [17]. Anastrozole is also an aromatase inhibitor [18]. Hormone sensitive types of cancers are stimulated by the female sex hormones such as estrogen and progesterone [19]. Being used as a hormone modulator, anastrozole is also frequently used in the treatment of pubertal gynecomastia [20]. Taking into consideration the importance of the drug, detailed investigations on anastrozole protein interactions were carried out employing fluorescence, dynamic light scattering, and molecular docking methods. Such knowledge on the mechanism of interaction between the anastrozole and plasma protein is of importance in understanding the pharmacodynamics and pharmacokinetics of anastrozole and anastrozole-designed new therapeutic drugs [21]. Drug binding to proteins may influence transportation, absorption, metabolism, and excretion of drugs [22]. Proteins are flexible molecules and ligand binding can affect the protein hydrodynamics and function; these alterations can be harmful or useful [23–26]. It becomes important to look at different aspects of drug-protein interactions when designing the dosage of the drugs spatially in a multidrug therapy or treatment in comorbid conditions, where the picture can be more complicated; protein binding of drugs not only affects drug pharmacokinetics but it can also affect the protein function. Here in the present study, the drug binds to the model protein and induced conformational alterations are reflected in the decreased catalytic function of the protein in the presence of the drug [27–30]. The interaction of anastrozole with the protein has been investigated under simulated physiological conditions using different optical techniques. Such studies can be extended to other important transporter proteins to give a more generalized view [31–33]. Investigation of interaction of anastrozole with HL assumes importance in life sciences, chemistry, and clinical medicine. The measures are of particular interest in clinical and pharmaceutical assessment.

## 2. Materials and Methods

Human lysozyme and anastrozole were products of Sigma-Aldrich. All other reagents used were of analytical grade.

### 2.1. Sample Preparation

All experiments were carried out in 20 mM sodium phosphate buffer of pH 7.4. HL was extensively dialyzed in buffer. Protein stock solutions (2.5 mg/ml) were prepared. The concentration of native protein in 20 mM phosphate buffer was determined using a double-beam UV-visible spectrophotometer (Cary 60, Agilent Technologies), the value of extinction coefficient used is *ε*_M_ = 37970 M^−1^·cm^−1^ at 280 nm [34]. 5 mg/ml anastrozole solution was prepared by weight/volume (w/v) in absolute alcohol [35].

### 2.2. Fluorescence Measurements

Cary Eclipse Fluorescence Spectrophotometer with thermal controller (Agilent Technologies) was used to measure the fluorescence emission spectra of HL. Emission spectra were recorded in the range of 300–500 nm with the excitation and emission slit width of 5 nm and 10 nm, respectively. Anastrozole is titrated into 5 *μ*M HL so as to obtain the range of drug-to-protein molar ratio of *d*/*p* = 0 to *d*/*p* = 10. Drug titrations were performed at 310 K, emission spectra were recorded, and data were plotted at 340 nm. The decrease in fluorescence intensity at 340 nm was analyzed according to the Stern–Volmer equation (equation (1)) [36, 37].

(1) $$\begin{array}{c} \frac{Fo}{F}=\text{Ksv}\left[Q\right]+1=\text{kq}\tau_{0}\left[Q\right]+1, \end{array}$$

where *Fo* and *F* are the fluorescence intensities in the absence and presence of quencher (anastrozole). Ksv is the Stern–Volmer quenching constant, kq is the bimolecular rate constant of the quenching reaction, and *τ*_0_ the average integral fluorescence life time biomolecules without quencher, which is ∼10^−8^ sec.

Binding constants and number of binding sites were obtained from modified Stern–Volmer equation (equation (2)) [38].

(2) $$\begin{array}{c} \text{Log }\left(\left(\frac{Fo}{F}\right)-1\right)=\log\text{Kb}+n\;\log\;\;\left[Q\right], \end{array}$$

where *Fo* and *F* are the fluorescence intensities in the absence and presence of quencher (anastrozole), Kb is the binding constant, and *n* is the binding stoichiometry. Gibbs–Helmholtz equation (equation (3)) was used for calculating thermodynamic parameters of ligand binding [39, 40].

(3) $$\begin{array}{c} \Delta {}^{G^{{}^{\circ}}}=-RT\;\ln\text{Kb,} \end{array}$$

where Kb is the binding constant at temperature *T*, ∆*G*° is free energy change, *R* is a gas constant (1.987 cal·mol^−1^·K^−1^) [41], and *T* is the absolute temperature (K).

### 2.3. Dynamic Light Scattering Measurements

DLS measurements were made using Zetasizer nano ZSP dynamic light scattering equipment (Malvern Instruments) equipped with a temperature-controlled sampler. DTS1070 folded capillary cells were used for size measurements. HL alone (10 *μ*M) and HL solution with anastrozole (100 *μ*M) was incubated for 2 hours before measurement. Measurements were then made, and hydrodynamic radius (nm) data were obtained using dynamics Zetasizer software provided with the instrument.

### 2.4. Molecular Docking

Anastrozole docking to HL was performed using 4.2.6 software (http://autodock.scripps.edu) [42–46]. Crystal structure of HL was obtained from Brookhaven Protein Data Bank PDB Id (1REX) and the coordinate 3D SDF file of anastrozole (CID 2794) was obtained from PubChem compound search. During docking calculations, all torsional bonds of anastrozole were set free to obtain possible conformation of the drug that binds to the protein, whereas HL was held rigid, and polar hydrogen was added using autodock tools. Grid size was set so as to cover all active site residues, and grid size was set at 126, 126, and 126 along *X*, *Y,* and *Z* axes. Auto dock rigid parameters were selected for protein chains and the ligand was allowed for flexible rotation. Lamarckian genetic algorithm (LGA) with GA population size 150 and maximum number of 2500000 energy evolutions were selected; the lowest energy conformation of the largest cluster of every docking simulation was extracted and analyzed. Best solution based on docking score was retained for further analysis and the docked conformation is visualized with Discovery Studio 3.5 Visualizer.

### 2.5. Muramidase Activity

Rate of loss of turbidity of *M. lysodeikticus* suspension was obtained using the absorbance value at 450 nm. 2 *μ*M HL was incubated with various molar concentrations of anastrozole at 298 K for two hours and then 10 *μ*L from each of these solutions was added to 2990 *μ*L of suspension of 0.3 mg/ml *M. lysodeikticus* in a quartz cuvette placed in the cuvette holder maintained at 298 K in the cuvette holder of Cary 60 double-beam UV–Vis spectrophotometer (Agilent Technologies). Decrement in absorbance values at 450 nm with time was monitored and used for the activity calculations [5]. Data obtained with the HL alone were assumed to be 100% and data obtained in the presence of anastrozole were organized with respect to the value obtained with HL alone that is considered as a reference. IC50 values are calculated using online software (https://www.aatbio.com/tools/ic50-calculator).

## 3. Results and Discussion

### 3.1. Fluorescence Spectroscopy

**Figure 1:**
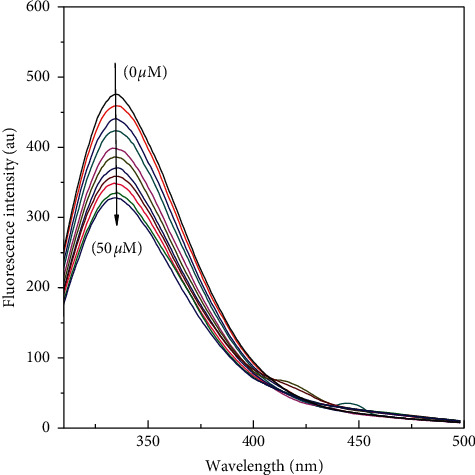
Fluorescence quenching of HL induced by anastrozole at 310 K. The concentration of HL was 5 *μ*M and the concentration of anastrozole varied from 0 to 50 *μ*M. The intrinsic fluorescence of the protein was measured in 20 mM sodium phosphate buffer, pH 7.4 at 310 K upon excitation at 280 nm.

To assess the binding considerations to protein molecule, steady state fluorescence quenching measurements were done and data were analyzed. The fluorescence spectroscopy technique has been widely used to study protein-ligand communications. Proteins have aromatic amino acid residues tyrosine, tryptophan, and phenylalanine. They serve as internal fluorophores. Tryptophan contribution in intrinsic fluorescence of proteins is maximum. Fluorescence quenching data for proteins can be engaged to obtain information on the protein-drug binding course. Ligand binding characteristics of ligand to the protein can be obtained. The interaction contrivances can be worked out using the fluorescence quenching data of the protein. In the present study fluorescence emission intensity of HL decreased proportionally with increasing drug concentrations (Figure 1).

**Figure 2:**
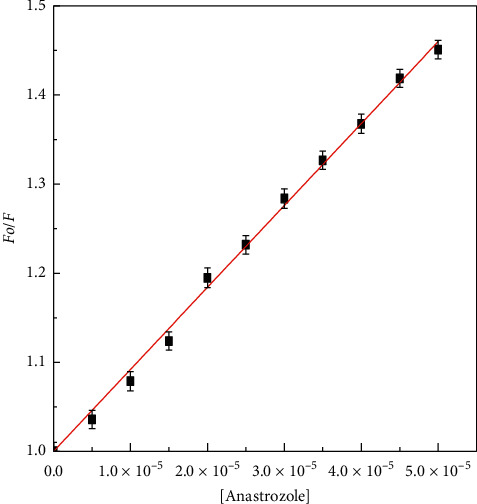
Stern–Volmer plots for fluorescence quenching of HL induced by anastrozole at 310 K. The concentration of HL was 5 *μ*M and the concentration of anastrozole varied from 0 to 50 *μ*M. The intrinsic fluorescence of the protein was measured in 20 mM sodium phosphate buffer, pH 7.4 at 310 K upon excitation at 280 nm.

There is sizeable decrease in fluorescence intensity of HL upon the titration of anastrozole. During the titration, the emission maxima remained unmoved. The fluorescence quenching data was analyzed according to the Stern–Volmer equation (equation (1)), and the bimolecular quenching constant for the interaction was calculated to be of the order of 10^11^ (Table 1) which is greater than the maximum collision quenching constant value obtained for various biomolecules 2 × 10^10^ M^−1^S^−1^ Moreover, Stern–Volmer quenching constant values obtained from linear Stern–Volmer plots are shown in Figure 2.

**Figure 3:**
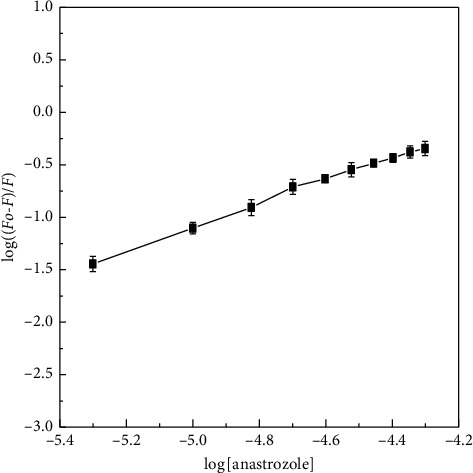
Modified Stern–Volmer plots for fluorescence quenching of HL induced by anastrozole at 310 K. The concentration of HL was 5 *μ*M and the concentration of anastrozole varied from 0 to 50 *μ*M. The intrinsic fluorescence of the protein was measured in 20 mM sodium phosphate buffer, pH 7.4 at 310 K upon excitation at 280 nm.

Fluorescence quenching records were analyzed according to the modified Stern–Volmer equations (equation (2)and binding constant and binding stoichiometry for the interaction of anastrozole was calculated with the slopes and intercept of the linear plots (Figure 3).

Binding constant is noteworthy in pharmacology and medicine. The binding constant of drug to proteins can significantly impact the transportation and absorption of drug molecules. Drug binding can affect the metabolism of drug compounds. The binding constant of HL anastrozole interaction was found to be near the order of 10^4^ at 310 K; binding stoichiometry was calculated near to 1 for the binding of anastrozole to HL at the temperature studied. Binding process of ligands to macromolecules involve many interactive forces including hydrogen bonding, hydrophobic interactions, van der Waals forces, and electrostatic interactions. Although individually these forces are weak, cumulatively these forces have huge impact on the behavior and function of ligand-protein interaction process. Thermodynamic calculations were performed using linear first law of thermodynamics and values for standard Gibbs free energy change were calculated using equation (3); the values are reported in Table 1.

We obtained negative value of Gibbs standard free energy change. The negative free energy change indicates spontaneity of interaction of anastrozole to HL. The decrement in free energy can be attributed to compensatory alterations in molecular hydrodynamics of protein upon ligand binding. Also, there occurs reformation of noncovalent interactions when ligand molecules entered the binding pocket in protein molecule.

### 3.2. Dynamic Light Scattering (DLS)

DLS is an important technique to study the changes in the molecular size of the protein in the solution. The hydrodynamic radius (*R*_h_) value at 298 K for HL at pH 7.4 was found to be 2 nm, which is unswerving with the earlier reports [47], *R*_h_ values were found to dwindle to 1.8 nm when HL was incubated with anastrozole (1 : 10) concentration. This shrink in *R*_h_ values may arise due to collapse of protein on drug binding. Ligand binding encourage rearrangement of conformational state and alterations in the protein upon anastrozole. Ligand binding may account for the decrease in hydrodynamic radii of HL and leading to molecular shrinkage reflecting in decrease in hydrodynamic radius of HL in presence of anastrozole. DLS measures overall total hydrodynamic size changes. Molecular shrinkage and expansion can be studied but specific region or domains cannot be studied. DLS results indicate size changes in the presence of anastrozole.

### 3.3. Molecular Docking

**Figure 4:**
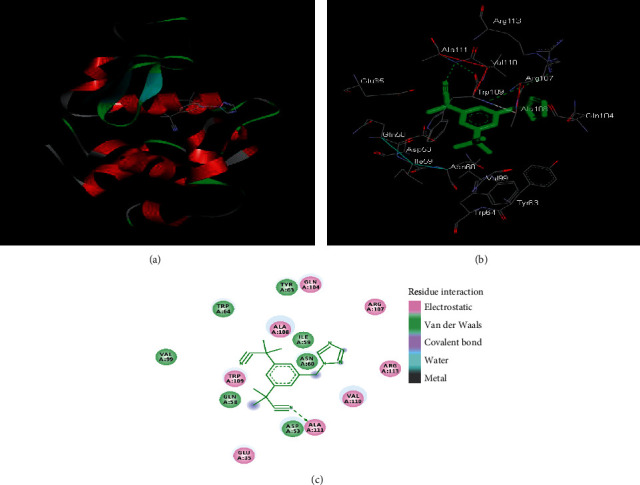
Molecular docking results of HL molecule with anastrozole. (a) Anastrozole is shown in a stick representation and HL is represented with ribbon model. (b) Detailed view of the docking poses of HL-anastrozole complex. (c) 2D plot of interaction of anastrozole with HL.

Molecular docking study was performed as described in section 2.4 to locate the binding region of anastrozole on the HL molecule. Molecular docking results suggested the anastrozole interacts with the HL via electrostatic and van der Waals interactions (Figures 4(a)–4(c)).

Amino acid residues found to interact with anastrozole are listed in Table 2 and there is involvement of polar amino acid residues in the interaction process while the free energy for the binding process from the docking results is −6.64 kcal·mol^−1^. Free energy change obtained from docking results although has same sign but values may differ because docking considers the backbone movements and overall fit of ligand into the binding pocket and protein remain rigid and the ligand is flexible. Changes in fluorescence emission are measured, and data are analyzed to obtain binding information. Fluorescence data are mainly the outcome of changes around fluorophores present in proteins.

As it is observed that amino acid residues are involved in binding of substrate to active site on the HL molecule, we further investigated the effect of anastrozole interaction on in vitro lytic action of lysozyme.

### 3.4. Muramidase Activity

**Figure 5:**
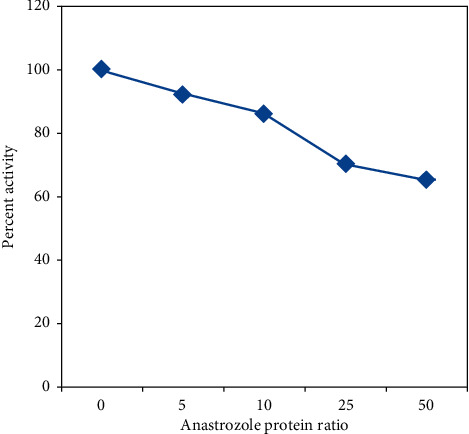
Activity results of HL with anastrozole; native HL muramidase activity is taken as 100%, at pH 7.4 and temperature of 298 K.

To test the lytic activity of HL in the presence of anastrozole, linear decrease of turbidity with time was used for the calculation of percentage activity in the HL samples with and without anastrozole. Enzymatic activity was found to decrease with increased anastrozole concentration and IC50 was found to be 10.78 (Figure 5).

It was found that the binding of the drug anastrozole brought about the conformational changes in the protein that negatively affected the lytic action of the protein. Decrease in the activity also signifies the effect of drug binding to proteins and far reaching effects on its structure and function.

## 4. Conclusion

The present study details the interaction of anastrozole with the model protein HL. Ligand binding effects on protein conformation and activity were studied. The binding course of anastrozole to HL is found to be spontaneous. Binding of anastrozole to HL induced hydrodynamic changes in HL and caused the molecule of HL to contract. Owing to topology alterations and involvement of amino acids near the catalytic site in drug binding, decreased in vitro lytic activity of HL was noticed. Drug-induced changes are plausible for the decreased catalytic efficiency of the protein in the presence of drug in in vitro studies. Anastrozole interacts with HL via hydrophobic and hydrogen bonding interactions. This study reflects the importance and multimodal effects of ligand binding to proteins. Therefore, understanding such interactions and drug binding parameters is imperative. The interaction of the HL with anastrozole can pave the way for the advancement of therapeutic and diagnostic strategies in conditions where disease is associated with protein function alteration and also in designing new molecules or studying drug repurposing.

## Figures and Tables

**Table 1:** Binding parameters of anastrozole interaction to HL in 20 mM phosphate buffer of pH 7.4 at 310 K temperature obtained and calculated from fluorescence quenching data.

*T* (K)	Ksv (×10^3^ M^−1^)	*R* ^2^	Kq (×10^12^ M^−1^s^−1^)	Kb (×10^3^ M^−1^)	*R* ^2^	*n*	Δ*G* (kcal·mol^−1^)	Δ*H* (kcal·mol^−1^)	Δ*S* (cal.·mol^−1^·K^−1^)

310	9.20	0.99	9.20	9.66	0.99	1.00	−5.65	−11.15	−19.30

**Table 2:** Molecular docking parameters obtained from HL-anastrozole binding.

Amino acid residues	Force involved	Δ*G* (kcal·mol^−1^)
Arg-113, Ala-111, Val-110, Glu-35 Trp-109, Arg-107, Ala-108, Gln-104 Gln-58, Val-99, Ile-59, Asp-53, Asn-60 Tyr-63, Trp-64	Electrostatic interactions and van der Waals interaction	−6.64

## Data Availability

Data used to support the findings of this study are available from the corresponding author upon request. No third party data were used in this work.
